# Pharmacogenomics to optimise psychotropic prescribing: a survey of mental health professionals’ perceptions, knowledge, and educational needs

**DOI:** 10.1038/s41397-025-00394-x

**Published:** 2026-01-20

**Authors:** Daniele Panconesi, Stephen Murtough, Marius Cotic, Noushin Saadullah Khani, Lauren Varney, Maria Richards-Brown, Rosemary Abidoph, Daisy Mills, Alvin Richards-Belle, Jazmin Molai, James Fenwick, Joanna Curwen, Matthew Allin, Alex Berry, Magdalana Barczyk, Stefania Bonaccorso, Rebecca Griffiths, Massimo Bernini, Ajai Kumar, Suruthy Senthilkumar, Yogita Dawda, Rajvinder Shokkar, Rosie Murdoch, Jamie Crane, Yousuf Rahimi, Myles Howard, Alison Welfare-Wilson, Agostina Secchi, Carmel Thomas, Bethany Pastor, Parveen Sharma, Georgy Pius, Rashad Nazir, Asif Mir, Jack Cheshire, Rhianne Bostock, Simon Gibbon, Pratima Singh, Chetan Shah, Sabrina Richards, Sai-Bo Cheung, Louise Rowe-Leete, Anita Jibero, Rebecca Cox, Philip Van Driel, Elvira Bramon

**Affiliations:** 1 Mental Health Neuroscience Department, Division of Psychiatry, University College London, London, UK; 2 North London NHS Foundation Trust, London, UK; 3 Central and North West London NHS Foundation Trust, London, UK; 4 Berkshire Healthcare NHS Foundation Trust, Bracknell, UK; 5 Kent and Medway NHS and Social Care Partnership, Maidstone, UK; 6 Greater Manchester Mental Health NHS Foundation Trust, Manchester, UK; 7 Nottinghamshire Healthcare NHS Foundation Trust, Nottingham, UK; 8 Hertfordshire Partnership NHS Trust, Hatfield, UK; 9 Surrey and Borders Partnership NHS Foundation Trust, Leatherhead, UK; 10 South West London and St George’s Mental Health NHS Trust, London, UK; 11 Somerset NHS Foundation Trust, Taunton, UK; 12 Institute of Cognitive Neuroscience, University College London, London, UK

**Keywords:** Genetic testing, Genetics research

## Abstract

A survey was conducted to determine attitudes, knowledge, and educational needs of mental health professionals regarding pharmacogenomics. We recruited 128 clinicians working in mental health in England, and we assessed their experiences using an adapted version of the “U‐PGx Clinician’s Questionnaire”. Responding clinicians had positive attitudes towards pharmacogenomics testing, although they lacked confidence in ordering and interpreting tests, for which most had never received any formal training. Only 6% of clinicians answered all 4 knowledge testing questions correctly, and barriers to clinical implementation included lack of familiarity and knowledge for several pharmacogenomics concepts, such as drug metabolism and genetics, as well as needing support from their working institution. Looking ahead, we found that accredited workshops and patient cases were preferred learning formats, and we suggest tailored education programmes to enable mental health professionals to apply pharmacogenomics in clinical practice.

## Introduction

Genetic factors play an important role in drug response, including both the development of adverse drug reactions – at times serious and potentially life-threatening – as well as therapeutic effectiveness [1]. By integrating pharmacogenomics at the point of care, it may be possible to minimise adverse drug reactions [2], maximise drug efficacy, reduce drug-drug interactions, and select medications based on patients’ genetic profiles. The discovery of genetic variants with clinical utility for prescribing has been documented over the past decade. As a result, the FDA has incorporated pharmacogenomics information into drug labels for 388 different medications, accounting for a total of 593 drug-gene interactions [3]. 36 of those medications (totalling 9.3%) are used in mental health treatments and are linked to 40 drug-gene interactions (Appendix 1); highlighting clear potential for pharmacogenomics interventions in mental health and psychiatry.

A growing body of evidence supports the use of genetic testing to inform drug prescribing, which is reflected in clinical guidelines developed by groups such as the Clinical Pharmacogenetics Implementation Consortium (CPIC) and Dutch Pharmacogenetics Working Group (DPWG). In the UK’s National Health Service (NHS), there are a few examples of this personalised approach to drug prescribing to prevent adverse drug reactions [4]. Testing for *DPYD* genetic variants is available prior to treatment with fluoropyrimidines in oncology [5], and neonatal mitochondrial genetic testing is available before treatment with aminoglycosides to prevent deafness [6]. However, the implementation of pharmacogenomics into clinical mental health practice faces major challenges in the UK and many countries worldwide [1–9].

Previous research around clinicians’ attitudes, acceptance, and knowledge of pharmacogenomics have suggested that accessibility to and lack of confidence in applying and interpreting genetic tests continues to limit their clinical use [10]. This highlights a need for standardised training programmes for medical and other clinical staff [7, 8].

Genetic testing is currently used to guide the treatment of several illnesses, especially in oncology and cardiology – areas of medical specialisation with the most extensive implementation of genetic testing to date. Meanwhile, translation of pharmacogenomics findings in mental health remains challenging [11], even though this is a medical specialty that could greatly benefit from pharmacogenomics implementation, for several reasons.

Firstly, mental illness has major impact both on a personal and societal level. It is estimated that mental health conditions cost the UK economy over £117.9 billion every year, which is 5% of the UK’s Gross Domestic Product. These costs are mainly associated with healthcare costs, loss of work productivity, and informal caregiver support [12]. Similarly, in 2010, mental health conditions cost the USA, 2.5 billion USD, and this is expected to increase significantly by 2024. And the cost of managing non-responders to antidepressant treatment is around 10,000 USD/year/patient greater than it is for managing responsive patients [13].

Secondly, the Sequence Treatment Alternatives to Relieve Depression trial [14] found that the response rate to initial antidepressant treatment was only 47%, and a systematic review concluded that non-responders to antidepressants were 15% more likely to attempt suicide, compared to 6% of patients with treatment-responsive depression and 1% of the general population [15]. In addition to this, as little as 28–33% of patients who take selective serotonin reuptake inhibitors (SSRI) achieve remission after initial antidepressant treatment [14]. Importantly, a recent meta-analysis found that individuals on pharmacogenomics-guided antidepressant treatment were 41% more likely to achieve remission, compared to patients on usual treatment [16].

Finally, regarding antipsychotic treatments, the Clinical Antipsychotic Trials of Intervention Effectiveness study [17] observed high discontinuation rates, and this is supported by a recent study synthesizing the evidence on this topic [18]. Furthermore, current antipsychotics have only marginal differences in efficacy, except clozapine. Therefore, choice of treatment is mostly empirical and guided by unique circumstances the individual [19]. Notably, promptness of treatment response seems to influence prognosis, as a minimal improvement after 2 weeks predicts a future non-response, with a specificity of 86% and positive predictive value of 90% [20].

Just et al. conducted a survey in 2017 among clinicians and researchers in different European countries and from various medical specialities to investigate their experience, perception, knowledge, and educational needs around pharmacogenomics [8]. Their findings were in concordance with previously published studies on this topic. Despite participants generally having a positive attitude to pharmacogenomics testing, uncertainty about its application and interpretation – due to limited experience and knowledge – posed a major barrier to its implementation in clinical practice. Furthermore, they observed that most respondents were open to engaging with education to fill the knowledge gap.

To our knowledge this is the first pharmacogenomics study to specifically focus on mental health professionals practising in England, and it aims to explore their views, experience, and knowledge, comparing these with Just et al.’s [8] findings as a benchmark.

We hypothesised that mental health professionals working in England would have similar views on pharmacogenomics compared with the cohort of clinicians examined by Just et al. [8]. However, we also theorised that some learning needs specific for mental health professionals might emerge.

## Materials and methods

### Ethical approval

This study was conducted in the context of the broader study, “Pharmacogenetics: Genetics and Environment in Mental Health Study (GEMS)” [21]. Ethical approval was provided by NHS Research Ethics Committee (19/LO/1403) and University College London ethics board (03/11/090). Formal inform consent was not required.

All participants were mental health professionals attending academic seminars, voluntarily took part in the survey and had capacity to consent; therefore, formal consent was not required given the nature of the study. Participants were informed that their answers would be anonymised. This study was performed in accordance with the relevant guidelines and regulations.

### Questionnaire design

The “Ubiquitous-Pharmacogenomics (U-PGx) Clinician’s Questionnaire” [8], a pre-existing survey of 42 questions developed by Just et al. in [8], was used. We made minor modifications to tailor the survey specifically for UK mental health professionals. Questions about age, country of practice, and an open query for comments were removed. The option to “prefer not to say” was included in the gender question. Three questions on general familiarity with pharmacogenomics were eliminated as they overlapped with other questions. We also adapted the question on medical specialities to focus on mental health subspecialties, and we amended the question about profession, by adding options for “nurse prescriber” and “scientist”. The adapted survey can be found in Appendix 2.

### Sample cohort and recruitment

Data were collected between 4^th^ May 2023 and 18^th^ July 2024. Inclusion criteria required participants to be mental health professionals practising in England, and all participants worked within three large mental health NHS trusts in London and one in Kent. They were invited to participate in the survey during educational events and conferences, which mental health professionals attend as part of their continued professional development. Mentimeter© and Microsoft Forms© were used to host the survey. Participants provided consent to take part, and their data were anonymised. In total, 128 participants were recruited and surveyed.

### Statistical analysis

Descriptive statistics were generated, and participants were analysed as a single group using R (v.4.3.2). Data were processed and figures presented as percentages (rounded to the nearest integer) using ggplot2 (v.3.4.3). Missing values for survey questions are reported in figure legends, and non-responders were discarded on a question-by-question basis. When integer values did not add to 100% (due to rounding errors), the Hare-Niemeyer method was used to allocate remainder portions via the coalitions package in R (v.0.6.24). When comparing findings with Just et al. [8], two proportion *z*-tests were performed using R. Percentage values and sample number were taken from the manuscript text by Just et al. [8]. After statistical analysis, *p*-values were adjusted for multiple comparisons using the false discovery rate method.

## Results

128 participants responded to the survey. All participants were practising clinicians in the NHS specialising in mental health, and their demographics and professional backgrounds are shown in Table 1. 78.9% were physicians (psychiatrists), and 10.2% were mental health specialist pharmacists. We included mental health specialist pharmacists and other mental health professionals as these groups are involved in mental health prescribing across NHS services. Moreover, we envisage these professions (alongside psychiatrists) to be involved in the ordering of pharmacogenomics tests when they become available. Additionally, 57% of the sample had more than 11 years of clinical experience, and nearly half of participants worked in a community-based setting (outpatients or ambulatory care; 46.1%), followed by participants based in a hospital inpatient setting (31.2%).

**Table 1:** Summary of survey participants.

Gender	% (No.)
Female	48.4% [62]
Male	47.7% [61]
Prefer not to say	3.9% (5)
Profession	
Pharmacist	10.2% (13)
Physician	78.9% (101)
Other	10.9% (14)
Primary Practice Setting	
Community-based Nursing Home	3.1% (4)
Hospital Inpatients	31.2% [40]
Outpatients or Ambulatory Care	46.1% [59]
Primary Care	0.8% (1)
Other	5.5% (7)
Non-responders	13.3% (17)
Work Experience in Years	
1	0.8% (1)
2–5	19.5% (25)
6–10	20.3% [26]
11–20	21.1% [27]
>20	35.9% [46]
Non-responders	2.3% (3)
	(*n *= 128)

Summary of clinicians who participated in the survey (*n* = 128). Percentages (rounded to 1 decimal place) and counts (included within brackets) are shown for each group. ‘Other’ in the ‘Profession’ category refers to mental health specialist nurse prescribers, scientists, physician associates, and those involved in providing pharmacogenomics clinical services; and ‘Other’ in the ‘Primary Practice Setting’ category refers to research and those working across multiple types of settings.

*PGx* pharmacogenomics.

### Analysis of mental health professionals’ experience and attitudes towards pharmacogenomics testing

**Fig. 1:**
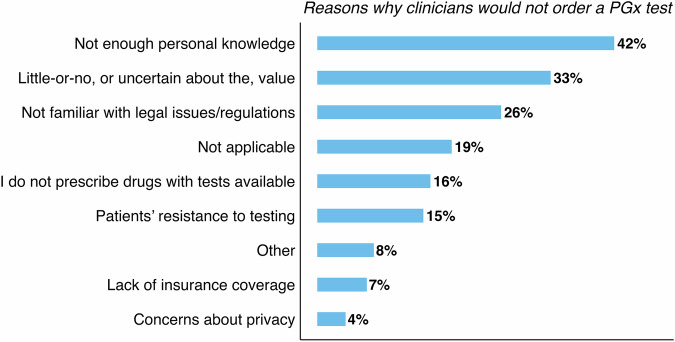
Reasons why clinicians working in mental health would not consider ordering a PGx test. Bar plot showing percentage of responses to survey question 32 (*n* = 124), which concerns reasons why clinicians would not order a PGx test (multiple answers were possible). Percentage values were rounded to the nearest integer and are shown. Missing values were removed prior to plotting and calculation of percentage values. PGx pharmacogenomics.

Most participants agreed that pharmacogenomics was relevant to their clinical practice (71%; Supplementary Fig. 1), although 82% had not ordered or recommended a pharmacogenomics test in the past year (Supplementary Fig. 2). We sought to understand the reasons why participants would not order or recommend a pharmacogenomics test (where participants could choose more than one answer), which included a lack of personal knowledge (42%), uncertainty about the value of pharmacogenomics testing (33%), and unfamiliarity with legal issues and regulations (26%) (Fig. 1). Some participants also reported that they do not prescribe drugs with pharmacogenomics tests available (16%) or that pharmacogenomics testing was not applicable to them (19%) (Fig. 1), which contrasts with the high number of mental health FDA drug labels with incorporated pharmacogenomics recommendations (Appendix 1) [3].

**Fig. 2:**
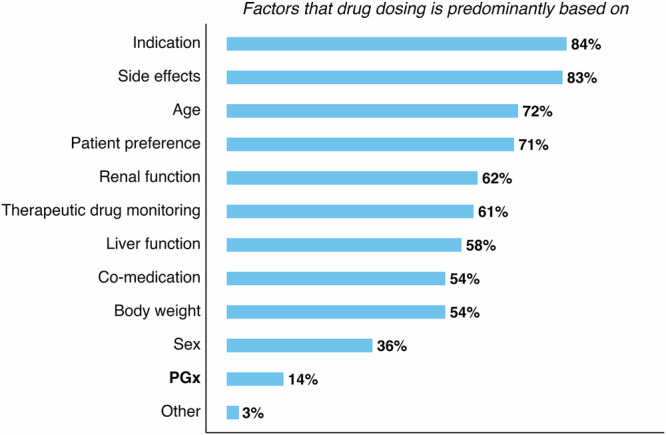
Factors that clinicians working in mental health consider for drug dosing. Bar plot showing percentage of responses to survey question 23 (*n* = 125), which concerns factors that clinicians predominantly base drug dosing on (multiple answers were possible). Percentage values were rounded to the nearest integer and are shown. Missing values were removed prior to plotting and calculation of percentage values. ‘PGx’ is shown in bold. PGx pharmacogenomics.

We then assessed which factors clinicians working in mental health considered for drug dosing (Fig. 2; multiple answers were possible). We found that clinical indication (84%), side-effects / adverse drug reactions (83%), and age (72%) were the most commonly cited reasons, while just 14% reported pharmacogenomics to be an important factor for drug dosing. This suggests a lack of awareness about the impact and relevance of pharmacogenomics in the dosing of commonly prescribed mental health drugs.

### Mental Health Professionals’ self-assessed knowledge and educational experience

**Fig. 3:**
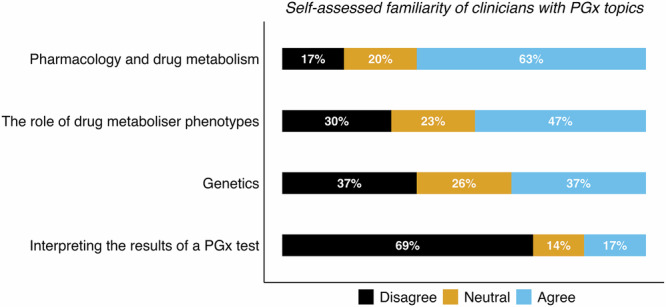
Self-assessed familiarity with PGx topics for clinicians working in mental health. Stacked bar plot showing the percentage of responses to survey questions, 8 (*n* = 117), 11 (*n* = 126), 14 (*n* = 125), and 17 (*n* = 126), regarding a participant’s familiarity with different PGx topics. Answers were scored on a 5-point Likert scale from ‘totally disagree’ to ‘totally agree’ (values of 0–4). For visualisation, ‘totally disagree’ and ‘disagree’ (values of 0 and 1) were aggregated to ‘Disagree’; ‘totally agree’ and ‘agree’ (values of 3 and 4) were aggregated to ‘Agree’; and ‘neither agree nor disagree’ (value of 2) was relabelled as ‘Neutral’. Percentage values were rounded to the nearest integer, and in cases where integers did not add to 100%, the Hare-Niemeyer method was used. Missing values were removed prior to plotting and calculation of percentage values. PGx pharmacogenomics.

**Fig. 4:**
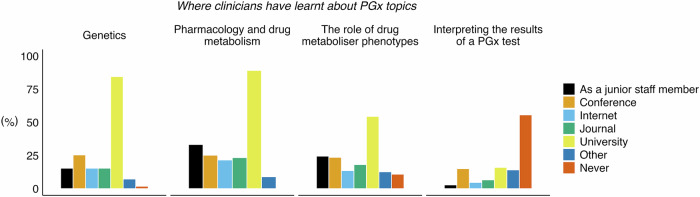
Places and sources where clinicians working in mental health have learnt about PGx topics. Series of bar plots showing percentage of responses to survey questions, 9 (genetics, *n* = 110), 12 (pharmacology and drug metabolism, *n* = 111), 15 (the role of drug metaboliser phenotypes, *n* = 110), and 18 (interpreting the results of a PGx test, *n* = 106), regarding where clinicians have learnt about PGx topics. For all questions, multiple answers were possible. Answer options are colour-coded and described in the figure legend. Missing values were removed prior to plotting and calculation of percentage values. PGx pharmacogenomics.

By self-assessment, most participants felt familiar with pharmacology and drug metabolism (63%), whereas under half felt familiar with the role of drug metaboliser phenotypes (47%) (Fig. 3). As for genetics, fewer respondents felt familiar (37%), and just 17% felt familiar with interpreting the results of a pharmacogenomics test (Fig. 3). We then assessed where the participants had previously learnt about these four pharmacogenomics topics (Fig. 4). We found that university was the most common source of knowledge for all topics, except for interpreting the results of a pharmacogenomics test, for which most participants (56%) reported they had never learnt about this. This highlights a potentially important knowledge gap in England (and the UK) for mental health professionals, which may act as a barrier when ordering and using pharmacogenomics tests in regular clinical practice.

### Pharmacogenomics factual knowledge

**Fig. 5:**
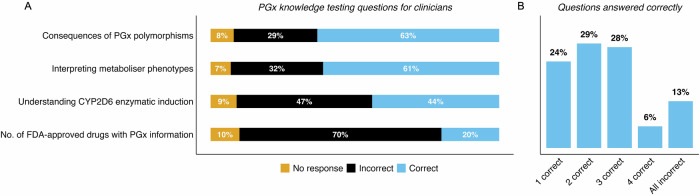
Assessing PGx knowledge in clinicians working in mental health. (**A**) Stacked bar plot showing how participants (as %; *n* = 128) answered questions, 26 – 29, which assessed their knowledge of PGx. Question names have been summarised into general topics for conciseness and ease of visualisation, and these are shown on the y-axis. One answer was correct for each question, and answers have been visualised as percentage values for ‘No response’, ‘Incorrect’, or ‘Correct’. CYP450 cytochrome P450 enzyme family. (**B**) Bar plot showing how many (as %; *n* = 128) questions participants answered correctly, and how many participants answered all questions incorrectly (including incorrect answers and no responses). For both charts, percentage values were rounded to the nearest integer, and in cases where integers did not add to 100%, the Hare-Niemeyer method was used. PGx pharmacogenomics.

We then tested participants’ knowledge of pharmacogenomics topics with 4 factual multiple-choice questions, each with 4 possible answers and only one answer being correct (Fig. 5). Overall performance in knowledge was calculated from 512 questions answered by all 128 participants (4 questions each). We found that 47.1% of questions were answered correctly (while the correct rate by chance would be 20%), 44.3% were answered incorrectly, and 8.6% of questions were not answered (data not shown). Answers for individual questions varied extensively. Most participants knew about the consequences of pharmacogenetic gene variants (What may be the consequence of a pharmacogenomic polymorphism?), as well as interpreting metaboliser phenotypes correctly (what does a poor metaboliser phenotype indicate?) (63 and 61%, respectively) (Fig. 5A). However, the other two questions were more challenging; a question about understanding CYP2D6 enzymatic induction (A person who is a poor metaboliser for CYP2D6 receives a medication that induces CYP2D6. What may be a consequence?) was correctly answered by just 44% of participants, and a question about how many FDA drug labels contain pharmacogenomics information was correctly answered by only 20% of participants (Fig. 5A). In addition, the overall level of pharmacogenomics knowledge among participants was low, with just 6% answering all 4 questions correctly, while 28% answered 3 questions correctly, 29% answered 2 questions correctly, 24% answered 1 question correctly, and 13% answered all questions incorrectly (Fig. 5B).

### Self-assessment of educational needs

**Fig. 6:**
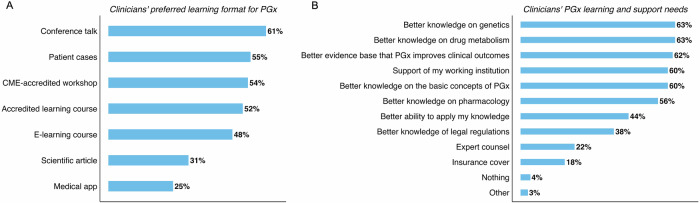
Clinicians’ PGx learning requirements and preferences. (**A**) Bar plot showing percentage of responses to question 35 (*n* = 105), which concerns clinicians’ preferred format for learning about PGx in the future. (**B**) Bar plot showing percentage of responses to question 33 (*n* = 116), which concerns learning and support needs to adjust therapy using PGx test results. For both charts, multiple answers were possible, percentage values were rounded to the nearest integer and are shown, and missing values were removed prior to plotting and calculation of percentage values. CME continuing medical education. PGx pharmacogenomics.

We asked participants whether they were confident in using the results of a pharmacogenomics test to make an appropriate adjustment to a patient’s drug therapy, to which 58% of participants disagreed, 17% were neutral, and 25% agreed (Supplementary Fig. 3). To address this knowledge and confidence gap, we asked participants how they would like to learn about pharmacogenomics (where multiple answers were possible). We found that conference talks (61%), patient cases (55%), and continuing medical education (CME)‐accredited workshops (54%) and accredited learning courses (52%) were preferred learning formats (Fig. 6A). We then asked what learning and support needs would help with this process, to which (among other answers) participants cited a better knowledge of genetics and drug metabolism (63% each), better evidence base that pharmacogenomics improves clinical outcomes (62%), and having the support of their working institution (60%) (Fig. 6B). Several answers to this question were reported by more than half of participants, stressing a need for more learning and support if pharmacogenomics is to be properly implemented in mental health clinical practice.

### Comparison of study findings with Just et al. [8]

In this study, we adapted a questionnaire that was previously used to survey healthcare professionals in European healthcare settings, which was published by Just et al. in 2017 [8]. A statistical comparison of our study findings with Just et al. (question by question) is shown in Table 2. For most questions, no significant differences were observed between the two survey’s findings, including questions concerning participant experience, knowledge, and learning preferences (Table 2). However, mental health professionals in our study reported a significant reduction in self-assessed familiarity with all four pharmacogenomics topics, compared with healthcare professionals surveyed by Just et al. (Table 2). This may reflect real differences in mental health clinical settings and is a factor that may need consideration before pharmacogenomics can be successfully included in regular clinical practice.

**Table 2:** Comparison of study results with Just et al.’s PGx survey [8].

Experience of, and attitudes toward, PGx	Study results, %	Just et al., %	Adjusted *p*-value
Is PGx relevant to my current practice?	70.9	84.3	ns
Have you ordered or recommended a PGx test in the past year?	18.3	34.3	ns
Is PGx a factor that you consider for drug dosing?	13.6	18.6	ns
**Self-assessed familiarity with PGx**			
Pharmacology and drug metabolism	63.5	85.8	**
The role of drug metaboliser phenotypes	47.2	75.7	***
Genetics	36.8	78.6	****
Interpreting the results of a PGx test	16.7	51.4	****
**PGx knowledge-testing questions**			
Correct	47.1	41	ns
Incorrect / no response	52.9	59	ns
**PGx learning preferences**			
Accredited learning course	52.4	60	ns
CME-accredited workshop	54.3	55.7	ns
E-learning course	47.6	47.1	ns
Patient cases	55.2	44.3	ns
Scientific article	31.4	44.3	ns

A table comparing this study’s survey results with Just et al.’s findings from 2017 [8]. Percentage values from Just et al.’s study were taken from the manuscript main text. Two proportion *z*-tests were used to statistically compare the two groups. Just et al. report a sample size of 70, which was used to calculate proportions, and sample numbers for this study are as reported in previous figures. Adjusted *p*-values were generated using the false discovery rate correction method, and these are shown as significance stars.

*ns* Not Significant.

**p <0.01.

***p <0.001.

****p <0.0001.

## Discussion

In this study, we investigated attitudes, knowledge, and educational needs of 128 mental health professionals practising in England about prescribing psychotropic drugs based on pharmacogenomics tests. We found that most mental health professionals had a positive attitude towards pharmacogenomics, although most did not feel familiar with ordering and interpreting the results of a pharmacogenomics test.

In addition, we found that most participants had learnt about key pharmacogenomics topics, such as genetics and drug metaboliser phenotypes, while at university, although most had not received any formal training for interpreting the results of a pharmacogenomics test. This highlights a knowledge gap that may need addressing to allow for safe implementation of pharmacogenomics in mental health services in England. Notably, participants’ performance in knowledge testing reflected these unmet educational needs, with just 6% answering all four questions correctly (with each question having four answers to choose form, and only one correct answer). We propose that specific training will provide mental health professionals with greater knowledge and confidence to order and interpret pharmacogenomics tests.

When asked what could be preventing participants from implementing pharmacogenomics testing in clinical practice, most required a better understanding of pharmacogenomics topics (including genetics and drug metabolism), as well as the support of their working institution. This suggests that two distinct strands may help to address these concerns. Firstly, tailored education programmes may be developed and offered to mental health professionals, with a focus on providing practical advice to aid interpretation of pharmacogenomics tests. Secondly, institutions (or more specifically, NHS trusts) should be aware that successful inclusion of pharmacogenomics into clinical practice may require their engagement and support. While beyond the scope of this study, NHS trusts that specialise in mental health should consider the types of support that are needed to ensure pharmacogenomics tests are ordered where it may benefit the patient.

Comparing our findings with Just et al. [8], we highlight that their cohort was composed of clinicians from a range of medical specialties participating in the “European pharmacogenomics clinical implementation project Ubiquitous Pharmacogenomics (U-PGx)” and therefore may be more experienced or interested in pharmacogenomics. By contrast, our cohort included a mixture of healthcare professionals, some of whom are involved in pharmacogenomics research. The proportions of physicians, pharmacists, and other professionals enrolled in their study were like ours.

Through a statistical comparison, shown in Table 2, responses from our cohort were not significantly different to Just et al. in questions about experience and attitudes toward pharmacogenomics; pharmacogenomics knowledge; and learning preferences. However, our cohort displayed a significant reduction in self-assessed familiarity with four pharmacogenomics topics, compared with Just et al. This may be specific to mental health services and suggests (as stated above) that attention should be given to developing and providing educational resources for this group of professionals. Moreover, this may also be specific to health services in England, which is where our cohort were drawn from. While no significant differences were observed in the number of correct answers to pharmacogenomics knowledge-testing questions, a lower self-assessed familiarity may result in fewer pharmacogenomics tests being ordered in mental health services. Indeed, while no significant differences were observed in proportions of participants ordering or recommending pharmacogenomics tests, numbers in our study were lower than Just et al. (18.3 and 34.3%, respectively; Table 2).

In addition, our research aligns with Chan et al.’s (2017) study [22], in which mental healthcare professionals were surveyed in Singapore. They found that most respondents (81%) believed pharmacogenomics testing could effectively identify suitable treatments, while 71% believed it could help with medication intolerance. However, like our study, only 46.4% felt confident in their ability to order these tests. Gender, profession (doctors versus pharmacists), and seniority were factors that significantly influenced responses. Additionally, 94.3% of respondents expressed concern about costs and 84.5% were concerned about the lack of clear guidelines for using pharmacogenomics in clinical practice. Indeed, 98.5% of respondents indicated a desire to learn more about the practical applications of pharmacogenomics, with education being preferred in a variety of formats.

Research has also indicated that clinicians face difficulties in incorporating pharmacogenomics information into their practice and are unsure of its impact [7–11]. This was further reinforced by a survey focusing on US nephrologists about their stance on pharmacogenomics testing [23]. Although they accepted the notion that genetic variations can influence course and treatment of chronic kidney disease, most medical professionals reported unease when discussing genetic results with patients whatever their opinion on pharmacogenomics testing may be. Nevertheless, physicians appeared more likely to utilise such tests when more aware of the supporting evidence.

Guo et al. [7] surveyed Chinese healthcare professionals about pharmacogenomics. They found that while 50% of physicians were unaware of the significance of pharmacogenomics in drug therapy, most were hopeful for its future in China, but regulations, sector standards, reporting standardisation, and a knowledge base must be implemented to make implementation of pharmacogenomics successful. These are similar to our study, given that improved knowledge base and institutional support were considered key factors for successful implementation of pharmacogenomics into regular mental health clinical practice.

St Savuer et al. [1] surveyed primary care physicians in the USA and discovered that only 30% made at least one change to their prescribed medication based on the pharmacogenomics alerts they received, while 53% did not find the alert reports useful. Around 45% of the respondents were unsure if they would use this alert system in the future. Furthermore, over half admitted feeling confused, irritated, frustrated or unable to find extra information when presented with a pharmacogenomics alert. Like our study, interpreting pharmacogenomics tests appears to be a key knowledge gap in several clinical settings.

Behr et al. [24] investigated the confidence and knowledge that US primary care and pain management healthcare providers have about pharmacogenomics. Most believed that pharmacogenomics could lead to better medication-related outcomes for patients, but at the same time did not feel prepared to apply the test results in their prescribing decisions due to a lack of experience with pharmacogenomics training and education, as well as inadequate familiarity with the United States Clinical Consortium (CPIC) and FDA resources for this purpose.

Our study was limited by the survey population consisting mainly of physicians, of whom general adult psychiatrists represented nearly half. This could influence the applicability of the results to different psychiatric subspecialties and general practitioners. The convenience sampling used could also limit its generalisability. Furthermore, participants were mainly drawn from two large mental health NHS trusts in London and recruited in the context of academic presentations, which are provided as part of a continuing professional development programme. These local meetings are mainly attended by psychiatrists, thus limiting a more multi-disciplinary sample.

The strengths of this study are that, to our knowledge, this is the first study specifically focusing on mental health professionals in England, aiming to explore their views and experiences around pharmacogenomics. A good degree of comparability with healthcare professionals from multiple clinical backgrounds was guaranteed by adopting the questionnaire by Just et al.

### Concluding remarks

This study suggests that mental health professionals have a positive attitude towards pharmacogenomics but face similar challenges to those of other healthcare practitioners when it comes to implementing pharmacogenomics in their clinical practice. It is evident that both a more tailored education program focused on interpreting pharmacogenomics tests and additional support from institutions are likely to be required for mental health professionals, so they can feel more comfortable ordering tests and making sense of results. To address some of these issues, experts from across the NHS have started collaborating with NHS England’s Genomics Education Programme to create GeNotes – genomic notes for clinicians – a ‘just in time’ educational resource about genomics and pharmacogenomics designed specifically for healthcare professionals [25]. We expect GeNotes to be one of many resources supporting pharmacogenomics-informed prescribing over coming years in UK mental health practice.

Research should aim to better characterise unmet educational needs and effective ways to address them, given that a growing body of evidence highlights a gap in knowledge preventing pharmacogenomics from being integrated into clinical practice, regardless of the medical specialisation. Further research is also needed to optimise the usability and implementation of pharmacogenomics test reports.

## Supplementary information

Supplementary Information

Fig.1

Fig.2

Fig.3

## Data Availability

The datasets generated during and/or analysed during the current study are available from the corresponding author upon request.
