# Supplementary material for: Pharmacogenomics to optimise psychotropic prescribing: a survey of mental health professionals’ perceptions, knowledge, and educational needs
Source: Pharmacogenomics J. 2026 Jan 20;26(1):2. doi: 10.1038/s41397-025-00394-x (PMC12819155; doi:10.1038/s41397-025-00394-x)

**Appendix 1**

**Table of mental health drug products with US FDA**

**pharmacogenomic information included in drug**

**labels.**

Data downloaded from the US FDA website. The full table

was filtered to include drugs listed in the ‘Psychiatry’

therapeutic area. Content last updated on 02/02/2024.

Data available at: https://www.fda.gov/drugs/science-and-

research-drugs/table-pharmacogenomic-biomarkers-

drug-labeling

**Appendix 2**

Link to **“Pharmacogenetic testing: survey for**

**clinicians”** [Pharmacogenetics testing: Survey for clinicians  – Fill in form](https://forms.cloud.microsoft/Pages/ResponsePage.aspx?id=_oivH5ipW0yTySEKEdmlwkA8VKFxWS1MhIaa64B-W_RUMVZOQlo0UTQ0RE5ROVNQQkxXWEUyWTgyRy4u)


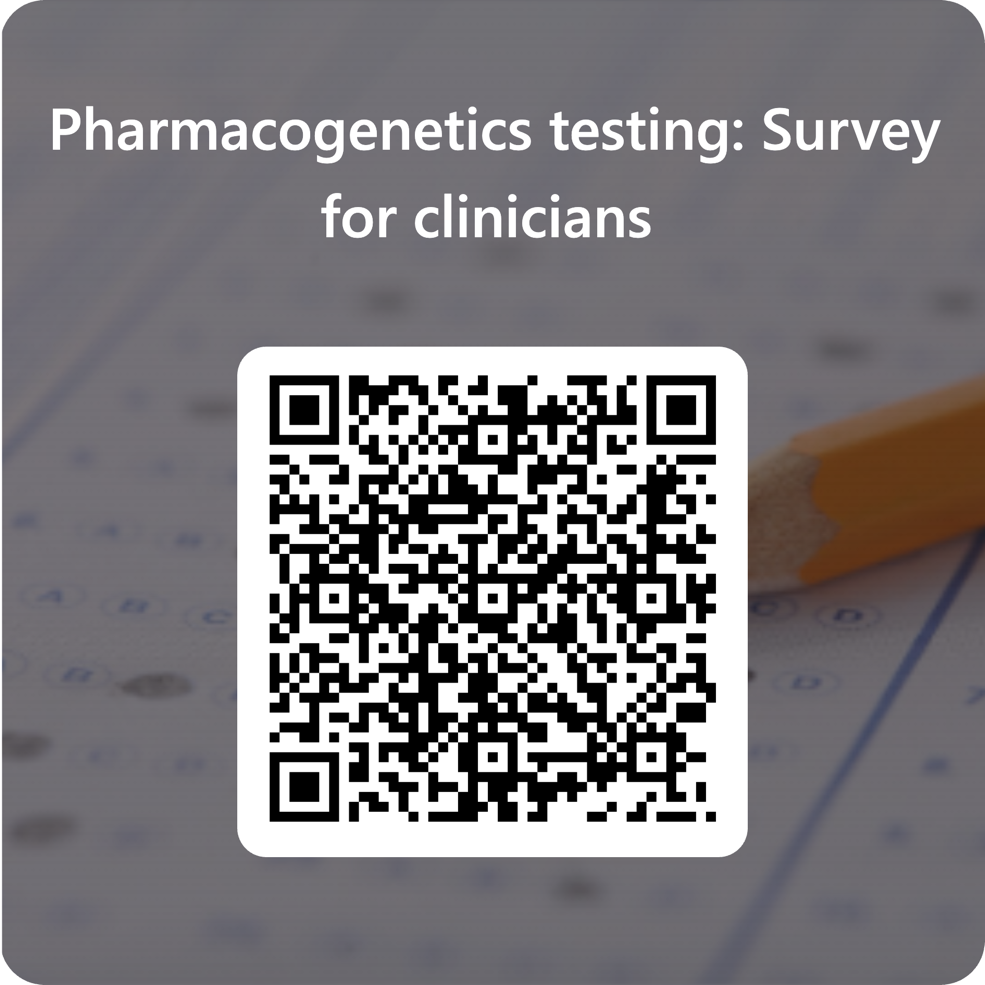

Supplement: Supplementary file 1 — Supplementary Information [file 41397_2025_394_MOESM1_ESM.docx]
