# Supplementary figures and images for: Pharmacogenomics to optimise psychotropic prescribing: a survey of mental health professionals’ perceptions, knowledge, and educational needs
Source: Pharmacogenomics J. 2026 Jan 20;26(1):2. doi: 10.1038/s41397-025-00394-x (PMC12819155; doi:10.1038/s41397-025-00394-x)

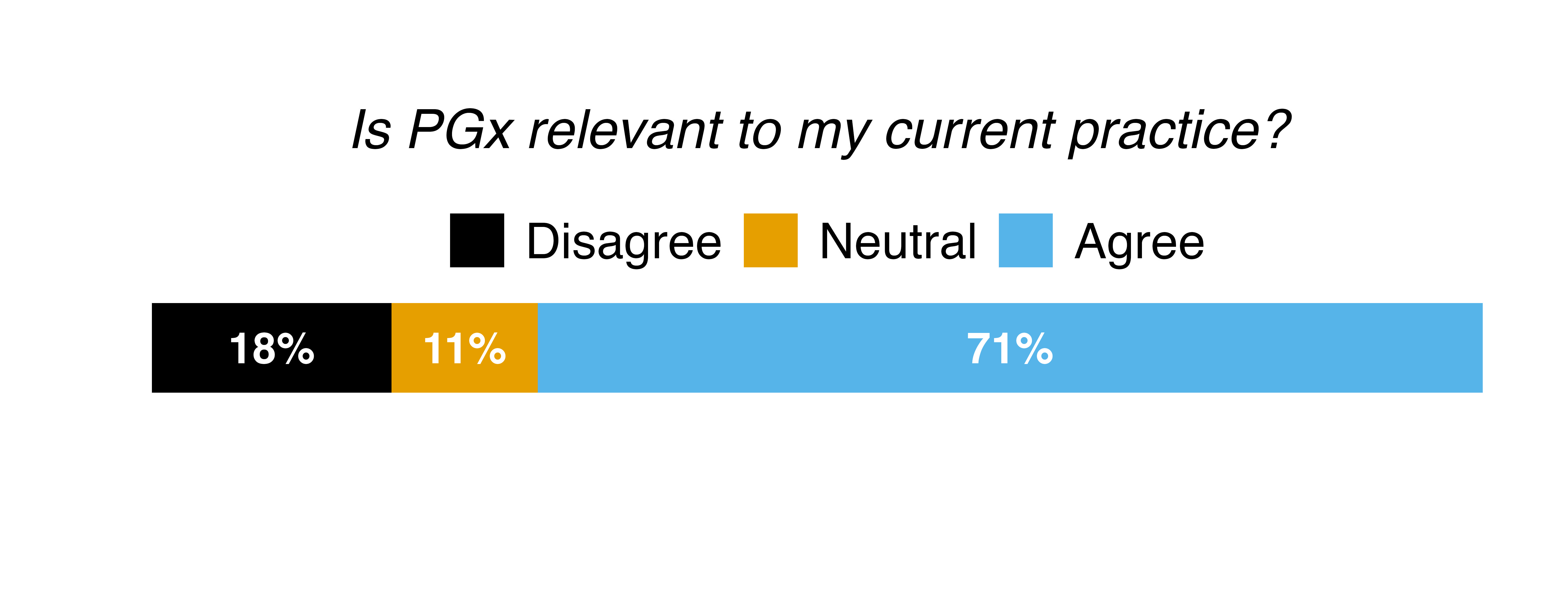

Supplement: Supplementary file 2 — Fig.1 [file 41397_2025_394_MOESM2_ESM.jpg]

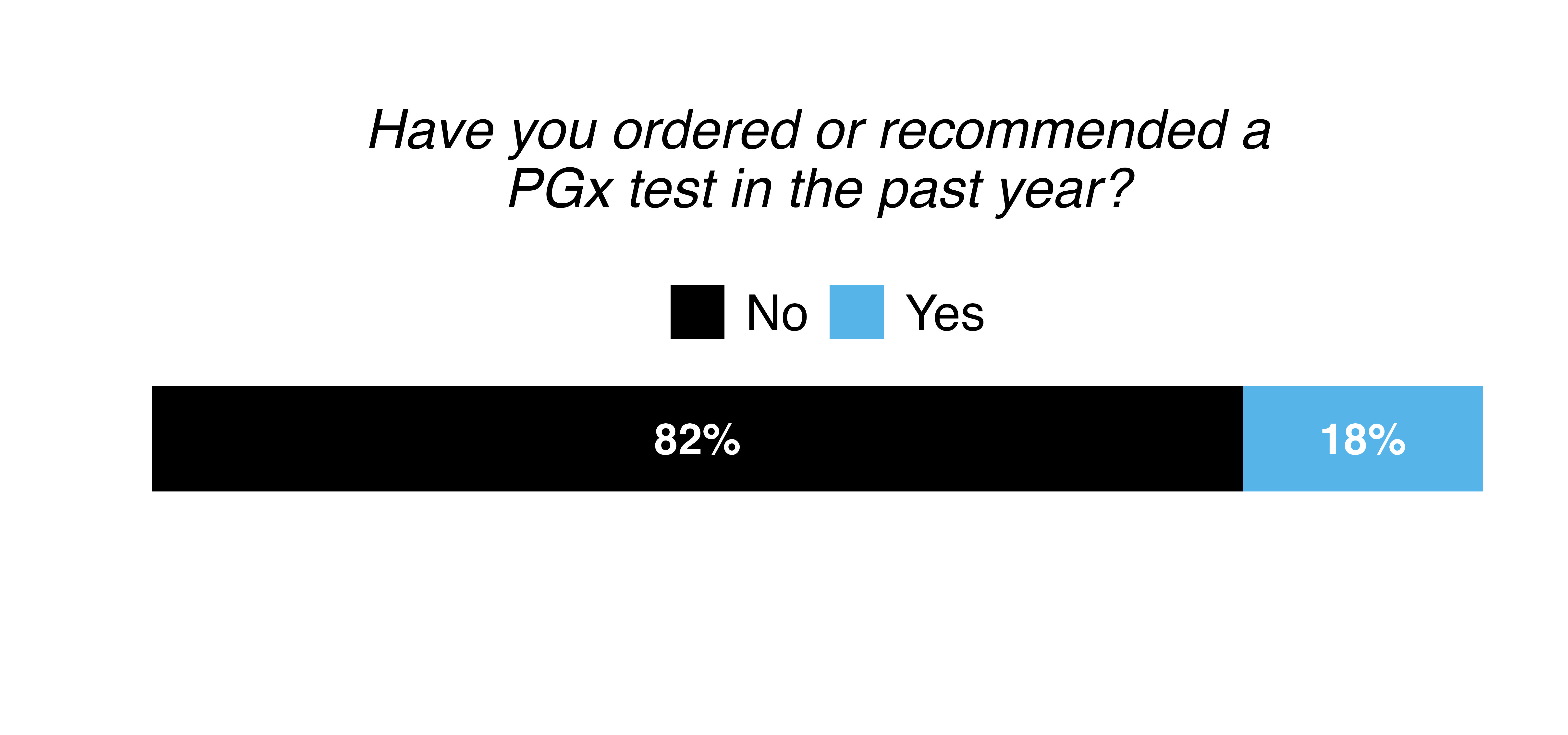

Supplement: Supplementary file 3 — Fig.2 [file 41397_2025_394_MOESM3_ESM.jpg]

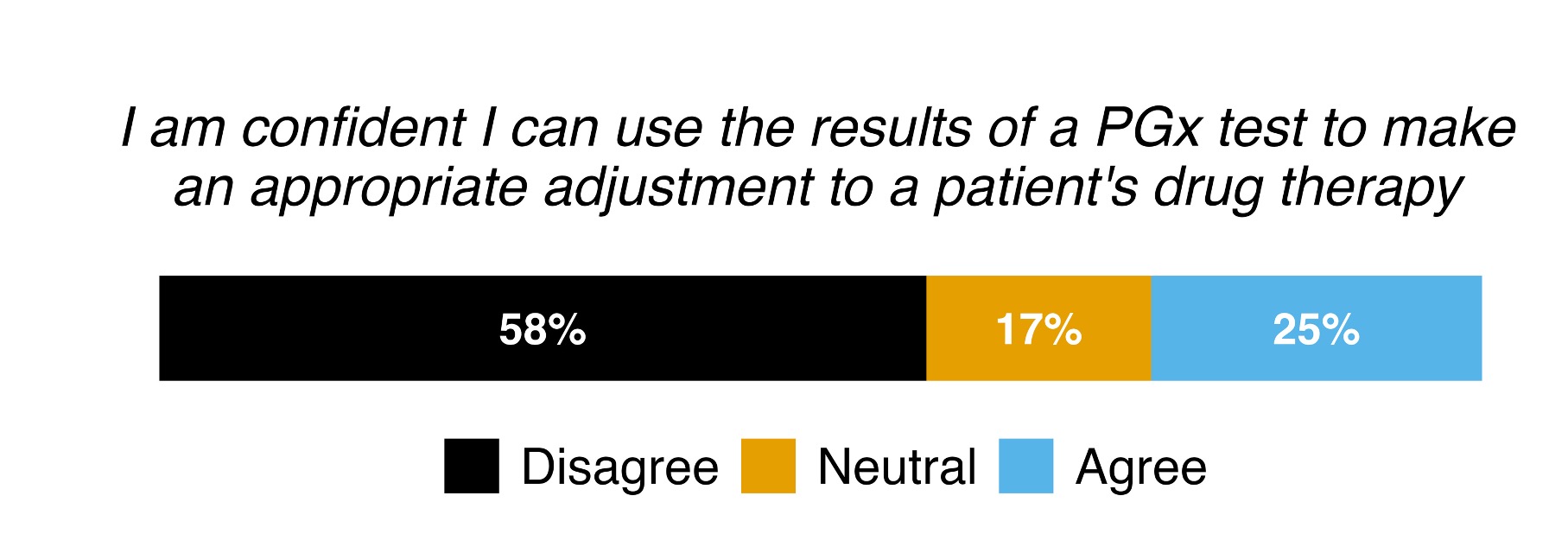

Supplement: Supplementary file 4 — Fig.3 [file 41397_2025_394_MOESM4_ESM.jpg]
